# Association of Serum Vitamin D and Immunoglobulin E Levels With Severity of Allergic Rhinitis

**DOI:** 10.7759/cureus.12911

**Published:** 2021-01-25

**Authors:** Nukhbat U Awan, Shahzada K Sohail, FATIMA NAUMERI, Shahida Niazi, Khalid Cheema, Samina Qamar, Syeda Fatima Rizvi

**Affiliations:** 1 Ear, Nose, Throat (ENT), King Edward Medical University, Mayo Hospital, Lahore, PAK; 2 Basic Medical Sciences, College of Medicine, University of Bisha, Bisha, SAU; 3 Pediatric Surgery, King Edward Medical University, Mayo Hospital, Lahore, PAK; 4 Department of Pathology, Sharif Medical & Dental College/Sharif Medical City Hospital, Lahore, PAK; 5 Pathology, King Edward Medical University, Lahore, PAK

**Keywords:** serum vitamin d levels, severity, allergic rhinitis, aria classification, serum immunoglobulin e levels

## Abstract

Objective

The aim of this study was to determine the association of serum vitamin D and immunoglobulin E (IgE) levels with the severity of allergic rhinitis (AR).

Methods

This case-control study was conducted at Mayo Hospital, Lahore, from June to September 2020 after obtaining ethical approval. Patients of AR were included and divided with the help of allergic rhinitis and its impact on asthma (ARIA) classification, into group A (cases), patients presenting with moderate to severe symptoms, and into group B (control), patients with mild symptoms, after treatment of AR. The mean difference between serum IgE and serum Vitamin D levels of both groups were compared by t-test. Association was determined by logistic regression and odds ratio.

Results

A total of 224 patients were included in the study, 112 patients in group A and 112 patients in group B. There were 106 (47.3%) female and 118 (52.7%) male. The mean age of patients in group A was 26.78± 8.92 years and in group B, it was 25.72±8.12 years. Mean serum vitamin D levels in group A were 16.24±6.7 ng/ml and in group B 26.92±35 ng/ml (p=0.0001). Mean serum IgE levels in group A were 383.69±154.86 IU/ml and in group B, they were 373.03±106.83 IU/ml (p=0.0001). Vitamin D deficient patients were 24 times more likely to develop moderate to severe AR disease.

Conclusion

This study showed that in moderate-severe AR, IgE levels are raised statistically as compared to mild AR and the deficiency of Vitamin D is associated with increasing severity of allergic rhinitis symptoms.

## Introduction

Allergic rhinitis (AR) is an inflammation of the nasal mucosa, mediated by immunoglobulin E (IgE), after exposure to different allergens [1]. Common allergens implicated in AR vary in different countries and include house dust mites, pollen, certain molds, occupational allergens, and animal danders [2]. Allergic rhinitis is thought to be the most common type of chronic rhinitis affecting up to 30%-40% of the population and its prevalence is increasing [3]. Moderate to severe AR affects the quality of life significantly due to poor performance at school and work, leading to sleep disturbance and social isolation [4].

Allergic rhinitis and its impact on asthma (ARIA) classification groups AR according to symptoms duration and severity. In the “Intermittent AR” symptoms duration is less than four days per week or four consecutive weeks while in “Persistent AR” symptoms last more than four days per week or more than four weeks. Symptoms are classified as ‘mild’ when there is no impairment in sleep and patients are able to perform daily routine activities and ‘moderate-severe’ if there is impaired sleep and symptoms are bothersome and affect daily activities [5].

Measurement of serum IgE levels can confirm the diagnosis of allergy and can be used as a prognostic marker to determine treatment outcome in allergic rhinitis, however, the role of total serum IgE levels in determining the severity of allergic rhinitis is uncertain and need further evaluation [6-8].

Similarly in literature, two contradictory hypotheses are reported since 1999 when Wjst and Dold pointed out a link between allergic diseases and vitamin D [9]. One hypothesis correlated that high serum vitamin D levels increased the prevalence of atopy and allergic diseases based on few studies noticing increased allergic conditions in infants taking vitamin D supplementation for rickets and born to mothers taking vitamin D supplementations during pregnancy; however, later on, other researchers found that vitamin D insufficiency increased risk of developing allergies and its severity [9-10]. Recent research work has shown that vitamin D receptors are expressed in various immune cells, suggesting the role played by vitamin D in the immune system and diseases mediated by this system [11]. The role of vitamin D deficiency in AR is thought to be associated with the immune-modulatory effects of derivatives of vitamin D [12].

Despite a lot of research work internationally, there is limited work locally to establish the association of low serum vitamin D levels and serum total immunoglobulin levels with severity of allergic rhinitis symptoms. We conducted this study to address this gap and to determine the association between the severity of allergic rhinitis symptoms, and serum IgE levels, and vitamin D levels.

## Materials and methods

A case-control study was conducted at Mayo Hospital, Lahore, from June 2020 to September 2020 after obtaining ethical approval from King Edward Medical University (824/RC/KEMU dated 09/11/2020).

Patients presenting with a history of atopy and recurrent sneezing, rhinorrhea, itching in nose and eyes, nasal congestion, nasal obstruction and postnasal drip (two or more symptoms), and typical physical findings on anterior rhinoscopy, such as pale, bluish tinged nasal mucosa, and the allergic salute was classified according to ARIA classification into mild (control), and moderate to severe allergic rhinitis patients (case) and were included in the study, after taking informed consent, using a purposive, non-probability sampling technique. The sample size of 224 patients (112 patients in each group) was estimated using a 5% level of significance, 90% power with expected mean serum vitamin D levels of 14.8±7.4 ng/ml in the case and 19.1±6.6 ng/ml in the control group [13].

In group A, patients with allergic rhinitis presenting with symptoms of moderate to severe AR were enrolled. In group B, patients with mild allergic rhinitis were taken as the control group only after treatment and control of symptoms. Blood samples from both groups were taken for serum IgE and serum vitamin D levels.

Serum vitamin D levels were measured by electrochemiluminescence immunoassay (ECLIA) by a hormone immunoassay analyzer. Levels of 30-100 ng/ml were considered normal, 20-29 ng/ml insufficient, and levels below 20 ng/ml were taken as deficient. Serum IgE levels were measured in IU/ml with the help of the Wizard gamma counter (PerkinElmer, Inc., Waltham, Massachusetts) and immunoCAP 100 (Thermo Fisher Scientific, Waltham, Massachusetts). Levels more than 150 IU/ml were taken as elevated.

Data were entered and analysis was done using the Statistical Package for the Social Sciences (SPSS) version 22. Frequency and percentages were measured for qualitative variables like gender, and mean ± standard deviation were calculated for quantitative variables (age, IgE levels, and serum vitamin D levels). For comparison of the mean difference of serum IgE and Vitamin D levels in both groups, an independent sample t-test was used. P-value <0.05 was taken as significant. Logistic regression was used to see the association between age, gender, deficient vitamin D levels and elevated IgE levels, and severity of AR. The odds ratio was calculated if the association was found to be significant (p-value <0.05).

## Results

A total of 224 patients were included in the study and divided into two groups according to the ARIA classification. One hundred and twelve patients were included as cases (group A) and 112 patients as control (group B). There were 106 (47.3%) female and 118 (52.7%) male patients. Overall mean age was 26.25±8.53 years, with a minimum of 10 years and a maximum of 52 years. Demographic characteristics of both groups are given in Table 1.

**Table 1:** Demographics detail of study population

VARIABLES	GROUP A (n=112)	GROUP B (n= 112)
Age (years)	26.78± 8.92	25.72±8.12
Male	59 (52.7%)	59 (52.7%)
Female	53 (47.3%)	53 (47.3%)

Overall mean serum vitamin D level was 21.58±7.56 ng/ml, with a minimum value of 8.81 and a maximum of 35.1 ng/ml. There were 97 (43.3%) vitamin D-deficient patients, 104 (46.4%) with insufficient levels, and only 23 (10.3%) with sufficient/normal vitamin D levels. Mean serum vitamin D levels in group A were 16.24±6.7 ng/ml and in group B were 26.92±3.5 ng/ml (p=0.0001).

Overall mean IgE levels were 378.36±132.84 IU/ml, with a minimum value of 103 and a maximum value of 740 IU/ml. Mean serum IgE levels in group A were 383.69±154.86 IU/ml and in group B, they were 373.03±106.83 IU/ml (p=0.0001) as seen in Table 2.

**Table 2:** Comparison of vitamin D and immunoglobulin E (IgE levels) in mild and severe cases of allergic rhinitis

VARIABLES	Group A	Group B	P-value
Serum Vitamin D level (ng/ml)	16.24±6.7	26.92±3.5	p=0.0001
Serum IgE level (IU/ml)	383.69±154.86	373.03±106.83	p=0.0001

Logistic regression revealed that deficient serum vitamin D levels were associated with severity of AR (p value=0.0001, 95% CI 0.01-0.13) while elevated IgE levels didn’t cause more severe AR (p value=0.9, 95% CI 0.99-1.01). The odds ratio was 24.11 (95% CI 7.6-76.3) in patients/control with deficient vitamin D levels to develop severe AR than with normal vitamin D levels.

## Discussion

A deficiency of vitamin D is a global health problem, affecting every segment of the population irrespective of age and gender and with a high prevalence in the third-world population [14].

In Pakistan, 50%-55% of the population is suffering from deficient vitamin D levels, and the most common causes of vitamin D deficiency include confinement to homes, decreased sunlight exposure, and improper diet [15]. In this study, 97 (43.3%) enrolled participants had deficient vitamin D levels; 90 of them were in group A. While only 23 (10.3%) had normal levels, 15 of them were in group B. Another study conducted in Pakistan reported deficient levels of Vitamin D in around 80% of enrolled participants, higher than 43.3% deficiency reported in this study [16].

Deficient vitamin D levels are known to be associated with severe AR and in our study, the mean vitamin D level in patients suffering from moderate-severe AR was 16.24±6.7 ng/ml and was statistically different from the control group (p=0.0001). This is comparable to the study of Anupam Malik et al. conducted in 2015 where mean serum vitamin D levels were 17.32±8.26 ng/ml [17]. Our findings are also comparable to the study of Sheeba F et al. conducted in 2019 in Pakistan reporting vitamin D levels of 14.8±7.4 ng/mL in severe AR patients [13]. The fact that deficiency of vitamin D results in more severe disease is also established by a previous study [16].

This study also showed that serum IgE levels in severe allergic rhinitis are significantly higher as compared to levels in patients with mild disease (p=0.0001). In this study, the mean serum IgE level in moderate-severe AR patients was 383.69±154.86 IU/mL. This is comparable to a study conducted in Turkey in 2018 by Mehmet Gokhan Demir, which included 256 patients and reported levels of 453.6±15.3 IU/ml in patients [18]. Our study findings are also comparable to the study of Osisi K et al. conducted in Nigeria in 2017, which revealed a statistically significant difference in serum IgE levels in the AR and normal population (252±60 IU/ml in patients and 106±117.95 IU/ml in healthy population) [19].

Serum IgE levels are considered a valuable diagnostic tool for suspected and confirmed allergic rhinitis patients [20]. However, the role of total IgE levels in assessing the severity of AR and in monitoring and tailoring specific therapy for AR is still debatable although the measurement of IgE levels is used as an indicator for anti-IgE immunotherapy in patients with AR. Immunotherapy with drugs such as omalizumab causes the downregulation of high-affinity IgE receptors on different immune cells, such as mast cells and basophils, reduces the levels of IgE, and helps in monitoring disease progression and treatment efficacy [21-22]. IgE levels can be used in selecting candidates for immunotherapy. Our study is limited, as it didn’t show that elevated IgE levels are associated with severity on logistic regression. The selection of patients treated for mild AR may have affected the levels, although we collected samples after the resolution of symptoms. A wider range of IgE levels with a minimum of 103 IU/ml and a maximum of 740 IU/ml may also be explained on the basis of selection criteria.

Vitamin D supplementation in AR patients with vitamin D deficiency can relieve the symptoms of AR and improve quality of life [23]. Similarly, one study established the fact that supplementation of vitamin D in deficient patients along with standard care significantly reduces the exacerbations of asthma [24].

This adequately powered, and well-matched study also documented the fact that patients who have deficient vitamin D levels are 24 times more likely to develop severe AR than those who have normal levels, thus supporting the use of vitamin D supplementation in AR patients. However, a wide confidence interval (7.6-76.3) may justify the need for future randomized control trials. Based on the findings, we recommend vitamin D supplementation in AR patients.

Limitation of the study

The limitation includes a possible selection bias, as patients with mild AR were included after treatment, and we were unable to demonstrate that elevated IgE levels are associated with increasing severity of AR.

## Conclusions

This study showed that in moderate-severe AR, IgE levels are raised statistically as compared to mild AR, and vitamin D deficiency is associated with increasing severity of allergic rhinitis symptoms.
